# Rational Design of PDA/P-PVDF@PP Janus Membrane with Asymmetric Wettability for Switchable Emulsion Separation

**DOI:** 10.3390/membranes13010014

**Published:** 2022-12-22

**Authors:** Jingjun Peng, Bhaskar Jyoti Deka, Shaodi Wu, Zhongyuan Luo, Jehad A. Kharraz, Wei Jia

**Affiliations:** 1 National Innovation Center for Advanced Medical Devices, National Institute of Advanced Medical Devices, Shenzhen 518110, China; 2 Department of Hydrology, Indian Institute of Technology Roorkee, Roorkee 247667, India; 3 Shanxi Engineering Research Center of Biorefinery, Institute of Coal Chemistry, Chinese Academy of Sciences, 27 South Taoyuan Road, Taiyuan 030001, China; 4 School of Energy and Environment, City University of Hong Kong, Tat Chee Avenue Kowloon, Hong Kong SAR, China; 5 Institute of Biomedical and Health Engineering, Shenzhen Institute of Advanced Technology, Chinese Academy of Sciences, Shenzhen 518110, China

**Keywords:** emulsion separation, Janus membrane, patterned interface, switchable wettability

## Abstract

Water pollution caused by oil spills or sewage discharges has become a serious ecological environmental issue. Despite the membrane separation technique having a promising application in wastewater purification, the membrane fabrication method and separation robustness have remained unsatisfactory until now. Herein, we developed a novel strategy, spacer-assisted sequential phase conversion, to create a patterned polyvinylidene fluoride@polypropylene (P-PVDF@PP) substrate membrane with a multiscale roughened surface. Based on that surface structure, the underwater oil resistance behavior of the P-PVDF@PP membrane was improved. Moreover, owing to the abundant active sites on the P-PVDF@PP surface, the polydopamine/P-PVDF@PP (PDA/P-PVDF@PP) Janus membrane could be readily fabricated via wet chemical modification, which exhibited excellent switchable oil–water separation performance. Regarding surfactant-stabilized oil-water emulsion, the as-prepared PDA/P-PVDF@PP Janus membrane also had robust separation efficiency (as high as 99% in the n-hexane/water, chloroform/water, and toluene/water emulsion separation cases) and desirable reusability. Finally, the underlying mechanism of emulsion separation in the PDA/P-PVDF@PP Janus membrane was specified. The as-designed PDA/P-PVDF@PP Janus membrane with high-efficiency oil–water separation shows potential application in oily wastewater treatment, and the developed fabrication method has implications for the fabrication of advanced separation membranes.

## 1. Introduction

In recent decades, increasing emissions from oily industrial wastewater, oily sewage discharges, or frequent oil spills have become a severe global threat to the eco-environment and human health [1,2,3,4]. Therefore, treating oil-contaminated wastewater, especially challenging emulsified oil–water mixtures, has developed into an urgent and necessary task [5]. Unfortunately, conventional separation approaches, such as the use of oil skimmers [6], oil sorbents [7], and gravity settling or centrifuges [8], are only applicable to the treatment of immiscible oil–water mixtures. They are ineffective in treating emulsified oil–water mixtures, especially emulsions with oil droplets smaller than 20 μm [9,10]. Although advanced chemical or electrostatic demulsification strategies have been exploited to separate stable emulsified mixtures [11], they suffer from high energy consumption and inevitable secondary pollution.

Membrane-based oil–water separation technology has been acknowledged as an advanced process for oil–water purification due to its simple fabrication, low pollution, high efficacy, and potential for scale-up [12]. Membranes with opposite wetting behavior (hydrophilic/oleophobic or hydrophobic/oleophilic) towards oil and water play an irreplaceable role in realizing the selective oil–water separation process. Due to dissatisfaction with the limited perm-selectivity of either oil or water, researchers further developed a new type of membrane with asymmetric wettability on each side, i.e., Janus membranes, to achieve the simultaneous separation of both oil-in-water and water-in-oil emulsions by simply switching the side facing the feed [13].

Creating and regulating asymmetry in membranes is the key issue for designing Janus membranes. Currently, most Janus membranes have been achieved by either asymmetric fabrication or asymmetric modification [14,15,16,17,18], among which, asymmetric modification is favored due to its greater flexibility in manipulating the membranes’ properties with various pristine structures. Nevertheless, although diverse strategies have been established to engineer the membranes’ surface properties, including single-faced photo-crosslinking, photo-degradation, single-faced coating, and vapor deposition [19], the asymmetric membrane surface-treatment technique remains challenging. The primary underlying issue with these methods is the porous nature of the membrane. Due to the capillary effect, the membrane is completely wetted during modification, causing a failure to construct asymmetry or yielding greater transmembrane resistance.

To overcome this problem, one common strategy is to introduce an additional interface on the surface of the membrane during the asymmetric modification process [20], which can protect the membrane from being entirely penetrated by the solution, thus enabling the desired single-sided modification. These interface-assisted strategies (gas–solid, gas–liquid, and liquid–liquid) were widely applied and proven to be efficient when tuning membrane asymmetry. For instance, Yang et al. presented a water-oil interfacial grafting method to construct a Janus membrane with an ultrathin hydrophobic layer, during which, the hydrolysate of the hydrophobic modifier is only soluble in oils rather than water [21]. This approach guaranteed the modification only occurred on the membrane surface, bringing about high separation efficiency and flux in the as-prepared membrane. Besides the liquid–liquid interface, Zheng et al. developed a Janus membrane by floating a dodecanethiol-Ag@PDA@nylon membrane on an L-cysteine solution, where a stable gas–liquid interface permits the occurrence of one-sided deposition on the connecting interface [22].

Another issue in the application of Janus membranes is delamination. In a typical fabrication process, the hydrophobic substrate is first modified with a low-surface-energy coating. Such a formed omniphobic surface usually weakens its interlayer interaction with the later coated with a hydrophilic layer, leading to less robust or unstable adhesion. Particularly, this delamination issue is exacerbated during liquid purification (e.g., oil–water separation and membrane distillation) since the hydrophilic layer is being hydrated [23]. Therefore, the pre-treatment process before asymmetric modification is considerable, which allows desirable interaction or bonding between the hydrophilic coating and substrate. For example, Zhu et.al. prepared a PVA@PAA@PVDF Janus membrane via sequential electrospinning/electrospraying in combination with the thermal treatment method, in which the thermal treatment provided a better interlamination interaction which was responsible for the stable interlayer structure [24]. Recently, mussel-inspired polydopamine (PDA) surface chemistry has attracted increasing attention in the membrane separation fields owing to its merit of surface-independent adhesion [25,26]. Polydopamine with hydrophilic functional groups, including quinone, catechol, and amine groups, is an ideal material for the preparation of the hydrophilic layer of Janus membranes. In addition, the bioinspired adhesivity of polydopamine permits a relatively stable interlayer interface for Janus membranes.

Inspired by the abovementioned research, we developed a novel spacer-assisted, non-solvent-induced phase conversion method to fabricate a PDA/P-PVDF@PP Janus membrane with excellent robustness for oil–water separation. The rhombic-shaped spacer endowed a multiscale roughened surface for the P-PVDF@PP substrate during the vapor and non-solvent-induced phase separation (VIPS and NIPS) process. In this way, the P-PVDF@PP substrate exhibited enhanced underwater oleophobicity and under-oil hydrophobicity. Notably, the P-PVDF@PP substrate with a multiscale roughened surface facilitated the subsequent PDA modification process via dopamine self-polymerization owing to the affluent active sites. Combined with the following gas–solid interface-assisted method (tape-assisted peeling-off), a PDA/P-PVDF@PP Janus membrane with anisotropic wettability was prepared. Based on the spacer-assisted method, the obtained PDA/P-PVDF@PP Janus membrane had high-efficiency and robust emulsion separation under the suction condition. This work provides a promising method for the fabrication of robust Janus membranes for switchable oil–water separation.

## 2. Materials and Methods

### 2.1. Chemicals and Apparatus

Non-woven polypropylene support (PP, Novatexx 2471) was purchased from Freudenberg-Filter (Weinheim, Germany). Polyvinylidene fluoride (PVDF, KYNAR HSV900) was provided by Arkema (Colombes, France) which was used as the membrane polymer. N, N-Dimethylacetamide (DMAc) (Purity ≥ 99.9%, Sigma-Aldrich, St. Louis, MO, USA) was utilized as the organic solvent for the preparation of the PVDF dope. The dopamine hydrochloride (DA, 98%), Oil red O, and Tris (hydroxymethyl) aminomethane hydrochloride (Tris-HCl) were supplied by Aladdin Reagent Co., Ltd. (Shanghai, China). Sodium dodecyl sulfate (SDS) was provided by Sigma-Aldrich. Span-80 was purchased from Tokyo Chemical Industry Co., Ltd. (Tokyo, Japan). Deionized water was produced from a Milli-Q^®^ water purification system (Merck Millipore, Burlington, MA, USA) and used in all the experiments.

### 2.2. PDA/P-PVDF@PP Janus membrane Fabrication

#### 2.2.1. Patterned and Hydrophobic P-PVDF@PP Substrate

**Figure 1:**
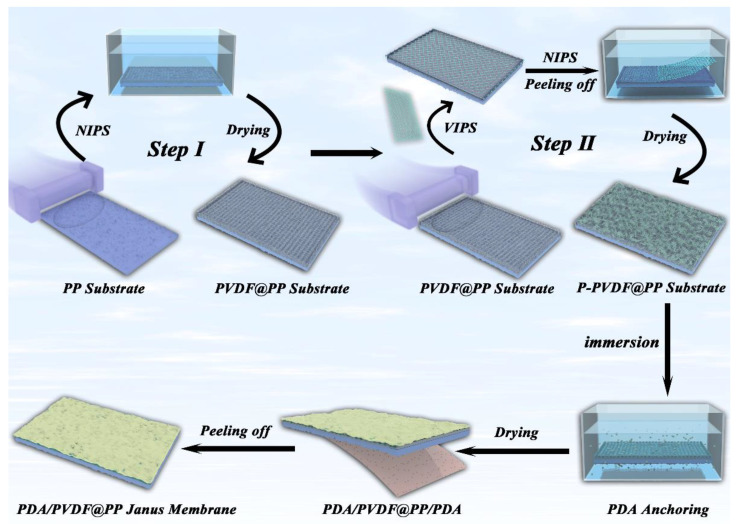
The construction process of the as-designed PDA/P-PVDF@PP Janus membrane.

The preparation process of the patterned and hydrophobic P-PVDF@PP substrate is shown in Figure 1 (Step Ⅰ and Step Ⅱ). First, the PVDF coating solutions were prepared by dispersing the PVDF powders (1.0 g and 1.2 g) into DMAc (9.0 g and 8.8 g) under vigorous stirring at 65 ℃. The PVDF concentration of the homogeneous solution was 10 wt.% and 12 wt.%, respectively [27]. Subsequently, the 12 wt.% PVDF solution (8 mL) was coated onto a clean PP substrate (300 × 200 mm) via a knife-coated device, with a wet film thickness of 230 μm. Finally, the stable PVDF@PP substrate was obtained via the non-solvent-induced phase separation (NIPS; deionized water) and drying processes, as displayed in Figure 1 (Step Ⅰ).

Next, the 10 wt.% PVDF solution (8 mL) was coated onto the prepared PVDF@PP substrate via the same knife-coated method; then, a rhombus mesh spacer (Figure S1) was immediately covered on the surface of the wet film and aged in a humid environment (25 °C, 80% RH) for 5 min. After that, the membrane with the spacer was immersed in a deionized water bath for 1 h (the spacer was removed after the first 1 min). Finally, the P-PVDF@PP membrane was obtained by drying it at room temperature for 48 h (Figure 1, Step Ⅱ). This imprinting and peeling-off step can introduce micro/nanostructured areas on a P-PVDF@PP membrane, increasing its active surface sites and surface hydrophobicity [28].

#### 2.2.2. PDA/P-PVDF@PP Janus membrane

To modify the PDA layer to prepare the PDA/P-PVDF@PP Janus membrane, the resulting P-PVDF@PP membrane (cut to 100 × 100 mm) was first immersed into a Tris-HCl buffer solution (400 mL) containing 2 mg·mL^−1^ dopamine (pH = 8.5) and subjected to an aging procedure at 60 °C for 12 h. Thereby, the hydrophilic PDA layer was anchored on both P-PVDF and PP sides via the dopamine adsorption and self-polymerization process. In the final step, the PDA/P-PVDF@PP Janus membrane was prepared by tape-assisted peeling-off (3M Scotch®, Saint Paul, MN, USA) of the unneeded PDA on the PP side.

### 2.3. Membrane Characterizations

The membrane surface and cross-sectional morphologies were observed via a field emission scanning electron microscope (FE-SEM, Hitachi SU5000, Tokyo, Japan) at a voltage of 10 kV and an emission current of 10.0 mA. The chemical states of the membrane surfaces were investigated via attenuated total reflection flourier transform infrared (ATR-FTIR) spectroscopy (Nicolet IR560, Madison, WI, USA) with the range of 650–4000 cm^−1^. According to the pressure step/equilibrate method, the pore size distribution was analyzed with a capillary flow porometer (Porometer POROLUX™1000, Nazareth, Belgium), during which, the dry membrane was firstly wetted with the wetting agent POREFIL and then sealed in a chamber.

The in-air WCA, under-oil water contact angle (UOWCA), and underwater oil contact angle (UWOCA) were all tested by using Kruss DSA25 EASYDROP (Hamburg, Germany). The UOWCA was measured by forcing the water droplet to contact the target surface of the Janus membrane with a bent needle. Before this, the membrane was first completely immersed with chloroform and then fixed facing downward. Likewise, the UWOCA was obtained by replacing the water droplet and chloroform with a soybean oil droplet and water, respectively, following the same procedure as that used for UOWCA. All the reported values in this work were presented by averaging a minimum of three tests [29].

### 2.4. Application in Oil-Water Separation

#### 2.4.1. Preparation of Oil-Water Mixtures and Oil-Water Emulsions

The immiscible chloroform/water and toluene/water mixtures were prepared with the same volume ratio of 1:1. Chloroform and toluene oils were dyed with oil red O before mixing with water for the facilitation of observations.

The surfactant-stabilized oil-in-water emulsions (o/w), including toluene-in-water emulsion (t/w), hexane-in-water emulsion (h/w), and chloroform-in-water emulsion (c/w), were prepared by diluting 1 mL of water into 100 mL of oil (toluene, hexane, or chloroform, respectively), during which, 0.1 g of Span-80 was added as the surfactant. Similarly, 100 mL water-in-oil emulsions (w/o), including water-in-toluene emulsion (w/t), water-in-hexane emulsion (w/h), and water-in-chloroform emulsion (w/c), were obtained by mixing 1 mL of oil (toluene, hexane, or chloroform, respectively), 0.1 mg of SDS surfactant, and a continuous phase of water for the rest. All the emulsions were magnetically stirred at room temperature for 24 h. After standing for several hours, the milky emulsions showed no demulsification, indicating their stability.

#### 2.4.2. Oil-Water Separation Test

The oil-water separation performance was evaluated using dead-end filtration apparatus driven by a vacuum pump. The membrane’s hydrophobic side faced the feed when treating the immiscible chloroform/water mixture and the water-in-oil emulsions. In contrast, the hydrophilic side faced upward when separating the toluene/water mixture and the oil-in-water emulsions. To test the reusability of the as-prepared Janus membrane, the membrane was simply rinsed with ethanol and water after each cycle. The permeation flux J (L·m^−2^·h^−1^) was calculated following Equation (1) according to previous studies [9,30].

*J* = *V*/(*A* × *T*), (1)

where *V* is the filtrate volume (L), *A* is the effective area (m^2^), and *T* is the operation time (h).

The separation efficiency (*ƞ*) for either the o/w or w/o separation process was calculated following Equation (2).

*ƞ* = (*C*_0_
*− C_p_*)/*C*_0_ × 100%, (2)

where *C_0_* and *C_p_* are the oil-water concentration (%) in the feed and filtrate, respectively (water concentration for the w/o separation and oil concentration for the o/w separation). The oil concentration was estimated with a total organic carbon (TOC) analyzer (TOC-VCSH, Shimadzu, Kyoto, Japan). Water concentration was measured using a Karl Fischer titrator (Byes6500, Bang. YES, Shanghai, China).

## 3. Results and Discussion

### 3.1. Fabrication of the PDA/P-PVDF@PP Janus Membrane

#### 3.1.1. Morphology of the Janus Membrane

**Figure 2:**
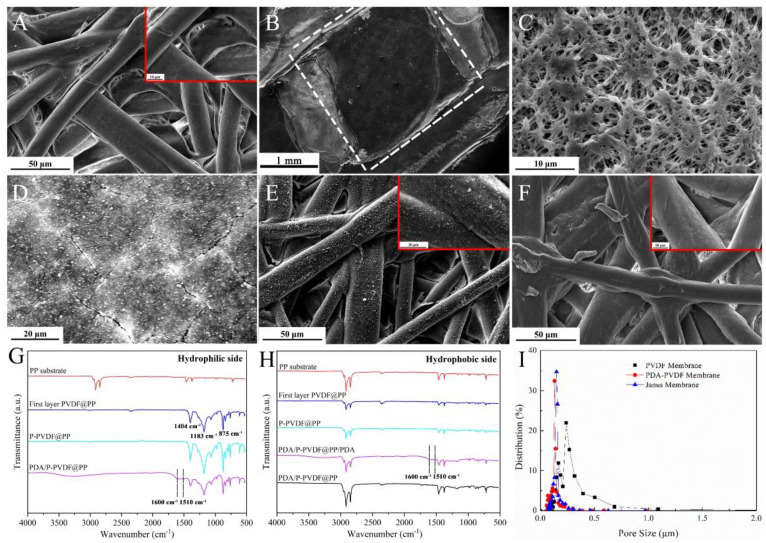
The SEM images (inserted are enlarged images) of (**A**) the PP membrane, (**B**,**C**) the P-PVDF@PP patterned surface, (**D**) the PDA/P-PVDF@PP Janus membrane surface, (**E**) the PDA/PP surface, (**F**) the PDA/PP surface after peeling-off; the ATR-FTIR spectra of (**G**) the hydrophilic side of the PDA/P-PVDF@PP, (**H**) the hydrophobic side of the PDA/P-PVDF@PP, (**I**) the pore size distribution of as-prepared membrane.

In this study, we developed a novel spacer-assisted, dual-phase separation (vapor and non-solvent) method to prepare Janus membranes with switchable oil-water separation capacities. The overall processes used for fabricating the PDA/P-PVDF@PP Janus membrane are illustrated in Figure 1. The pristine PP substrate had a relatively smooth surface that was composed of cross-linked fibers (Figure 2A). After sequential modification (Step Ⅰ and Step Ⅱ), the PP surface was coated with a PVDF layer consisting of interlaced fine cello silk subunits (Figure 2C). With the aid of a spacer, moreover, the patterned surface of the membrane was created which endowed a multiscale roughness micro/nanostructure for P-PVDF@PP (Figure 2B,C). This multiscale roughness texture could improve the surface’s hydrophobicity, protecting the membrane from complete infiltration during the polydopamine grafting or adhesion process. On the other hand, the cross-sectional images showed that the interlayer between P-PVDF and PP was blurry, suggesting the excellent compatibility of interlayer materials, which greatly enhanced the robustness and stability of the Janus membrane (Figure S2).

As displayed in Figure 2D, the surface microstructure of the PDA/P-PVDF@PP membrane with a significant change demonstrated the successful modification of PDA. During the reaction period, the PDA polymers rapidly aggregated throughout the PVDF surface, while they barely did on the PP side (Figure 2D,E). This may have been caused by the asymmetric surface roughness affecting the specific surface areas for anchoring PDA polymers. The multiscale roughness surface on the PVDF side provided abundant active sites to grow the PDA polymers rapidly. Inversely, the relatively smooth surface on the PP side slowed the growth process. Therefore, thanks to the asymmetric modification, the PDA polymer attached to the PP side could be easily peeled off to obtain the PDA/P-PVDF@PP Janus membrane (Figure 2E,F).

#### 3.1.2. Chemical Composition of the Janus Membrane

The chemical compositions of the PP substrate, P-PVDF@PP, and as-prepared PDA/P-PVDF@PP Janus membrane were further investigated via ATR-FTIR measurement. Regarding the P-PVDF@PP membrane, the ATR-FTIR spectrum displayed three strong characteristic absorption peaks at 1404 cm^−1^, 1183 cm^−1^, and 875 cm^−1^ (Figure 2G), which were assigned to the stretching vibration of −CH_2_, −CF_2_, and C−C, respectively, belonging to the characteristic peaks of the PVDF component [31,32]. For the PDA/P-PVDF@PP Janus membrane, the weak peaks located at 1510 cm^−1^ and 1600 cm^−1^ were ascribed to the deformation vibration of N−H and stretching vibration of C=O, while new broad peaks distributed on 3000–3500 cm^−1^ stemmed from N−H stretching and O−H stretching [33]. These results certify the successful modification of the PDA layers (Figure 2G). On the other hand, the P-PVDF@PP substrate also exhibited similar characteristic peaks to PP, implying the large-sized pore structure of the PP substrate only permitted the partial penetration of PVDF solution during the knife-coating process (Figure 2H). In addition, there was no noticeable peak change between the pristine PP substrate and the PDA/P-PVDF@PP Janus membrane, confirming that PDA was successfully removed via the tape-assisted peeling-off process (Figure 2H).

Simultaneously, the P-PVDF@PP membrane’s pore structure was regulated with the anchoring of PDA, which narrowed the distribution of pore diameter in the P-PVDF@PP membrane. As shown in Figure 2I, the pore size of the P-PVDF@PP membrane substrate primarily fell in the range of 0.20–0.24 μm while approximately shrinking to 0.14 μm with the modification of the PDA. Of note, the variation in the pore diameter distribution in the PDA/P-PVDF@PP Janus membrane was marginal after the peeling-off process (Figure 2I, blue curve). This result indicates that the hydrophilic PDA layer on the P-PVDF side was relatively dense. As a result, a synergistic structure existed in the as-prepared Janus membrane, i.e., the combination of the hydrophobic layer with large-sized pores and hydrophilic with small-sized pores. Such a synergistic structure for water-oil mixture separation can facilitate the water transport process while enhancing the transmembrane pressure of oil [34].

### 3.2. Janus Membrane Wettability

The switchable surface wettability of the Janus membrane is crucial for the realization of the efficient separation of w/o and o/w emulsions. Thereby, a series of contact angle measurements (in-air WCA, UOWCA, and UWOCA) were employed to comprehensively estimate the asymmetric wettability of the as-fabricated membrane. As shown in Figure S4A, the original PP substrate exhibited a moderately hydrophobic nature with an in-air WCA of about 129.1°. It is well known that a membrane surface’s hydrophobicity is determined by the surface’s topological structure and its intrinsic surface energy [35]. Hence, as expected, the hydrophobicity of the P-PVDF@PP membrane was substantially improved (in-air WCA increased to 136.1°) by introducing the rough micro/nanostructure into a low-energy surface (Figure S3).

**Figure 3:**
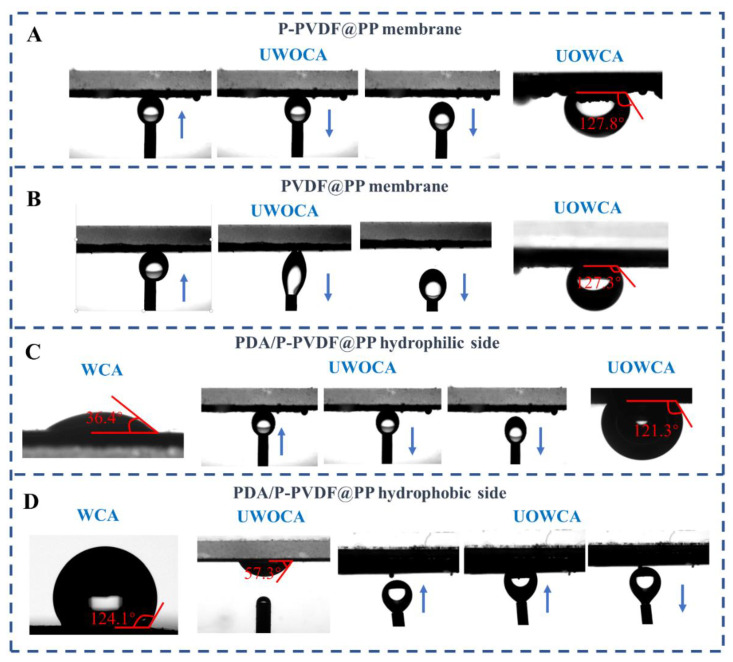
The UWOCA and UOWCA of (**A**) P-PVDF@PP membrane and (**B**) PVDF@PP membrane; the WCA, UWOCA, and UOWCA of (**C**) PDA/P-PVDF@PP hydrophilic side and (**D**) PDA/P-PVDF@PP hydrophobic side. All the arrows mean the direction of droplet motion.

It is worth mentioning that the P-PVDF@PP membrane with rough micro/nanostructure also displayed improved performance regarding the underwater oleophobicity. As disclosed in Figure 3B, during the relaxation process of the UWOCA test, the spherical oil droplet on the non-patterned PVDF@PP surface was drawn to the elliptical and partly adhered to the membrane surface. In contrast, the oil droplet on the P-PVDF@PP surface remained spherical even under squeeze conditions (Figure 3A). The parahydrophobic property of the P-PVDF@PP membrane, which means the hydrophobic surface with high water adhesion, may have been responsible for the enhanced underwater oil resistance [36]. Concerning the UOWCA tests, the result of P-PVDF@PP at about 127.8° was similar to the non-patterned PVDF@PP sample, in which both surfaces commendably trapped the oil which served as the barrier preventing the water from direct contact with the membrane surface (Figure 3A,B).

Once grafted with the PDA polymers, the P-PVDF@PP membrane surface turned hydrophilic with a low in-air WCA of 36.4° (Figure 3C) due to its excellent hydrophilicity [37]. Meanwhile, Figure 3C shows that the squeezed oil droplet under water could be quickly drawn back with ignorable distortion on the upside of the PDA/P-PVDF@PP Janus membrane, elucidating its excellent oil adhesion resistance. Predictably, the PDA/P-PVDF@PP membrane was under-oil hydrophobic (UOWCA = 127.8°) due to the immiscibility between oil and water (Figure 3C). With regard to the backside of the PDA/P-PVDF@PP Janus membrane, the in-air WCA was recovered to 124.1° after undergoing the peeling-off process, which was closer to the initial value, and thus demonstrated the successful removal of the PDA nanoparticles (Figure S4B and Figure 3D). Furthermore, as shown in Figure 3D, the underwater oleophilicity and under-oil hydrophobicity which performed on the backside of the PDA/P-PVDF@PP membrane showed the switchable oil-water separation capacity of the as-fabricated Janus membrane.

### 3.3. Oil-Water Separation Performance

**Figure 4:**
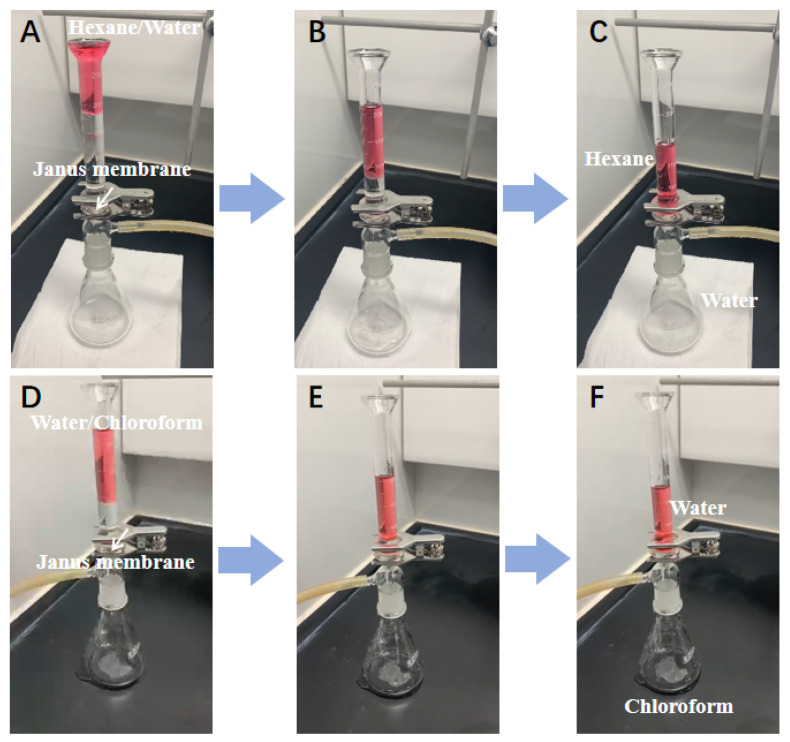
Optical photographs of (**A**–**C**) hexane (light oil)/water separation and (**D**–**F**) water/chloroform (heavy oil) separation process.

To gain insights into the switchable oil-water separation capacity of the well-designed PDA/P-PVDF@PP Janus membrane, a commercial dead-end filtration device with the membrane sealed in the middle was employed as the separation apparatus. As shown in Figure 4A–C, with the intervention of the PDA/P-PVDF@PP Janus membrane (the hydrophilic PDA side was face up), the immiscible light oil (hexane)/water mixture could be quickly separated under a moderate suction force from the pump. Similarly, the immiscible heavy oil (chloroform)/water mixture was also rapidly divided into two parts by switching the PP side face up, in which the chloroform permeated and passed through the Janus membrane while the water was obstructed and retained in the upper tube (Figure 4D,E). The separation efficiency was 99.95% for the water/hexane and 99.83% for the chloroform/water mixture, exhibiting remarkable separation efficiency.

**Figure 5:**
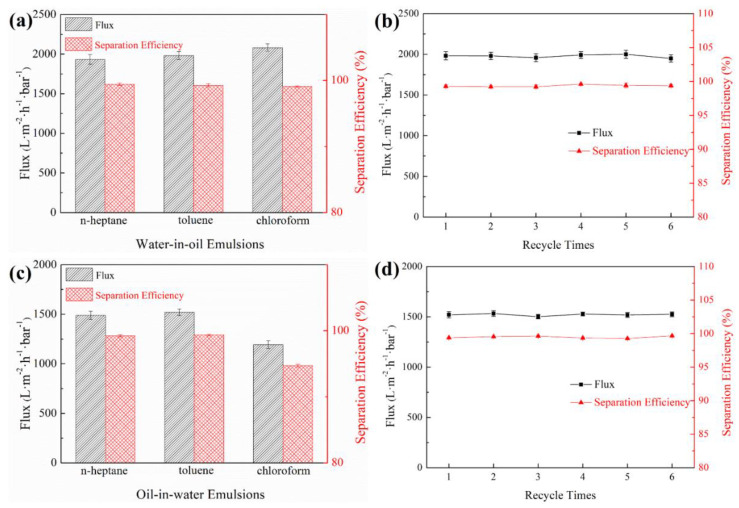
(**a**) Separation flux, the efficiency of different water-in-oil emulsions in as-prepared PDA/P-PVDF@PP Janus membrane; (**b**) the reusability of the PDA/P-PVDF@PP Janus membrane for the separation of the water-in-toluene emulsion; (**c**) separation flux, the efficiency of different oil-in-water emulsions in as-prepared PDA/P-PVDF@PP Janus membrane; (**d**) the reusability of the PDA/P-PVDF@PP Janus membrane for the separation of the toluene-in-water emulsion.

The oil-water separation performance for diverse w/o and o/w emulsions was comprehensively studied with the as-prepared PDA/P-PVDA@PP Janus membrane. The optical microscopy experiments were performed to evaluate the filtrates qualitatively. For the typical samples, with the aid of the Janus membrane, both o/w and w/o emulsions were clarified by filtering (Figure S5). In detail, the separation efficiency of water-in-oil emulsions could reach up to 99%, and the flux for the separation of w/h, w/t, w/c emulsions was 1950.34, 1980.54, and 2123.64 L·m^−2^·h^−1^, respectively. Further, for the separation of the oil-in-water emulsion, the PDA hydrophilic side needed to be oriented toward the emulsions, where the superior in-air hydrophilicity and underwater oleophobicity of the PDA layer guaranteed the satisfying oil rejection rates during the separation process. Therefore, the separation performances of the as-designed Janus membrane for the oil-in-water emulsions were desirable, and the separation efficiency of h/w, t/w, and c/w emulsions was 99.61%, 99.53%, and 95.07%, along with the flux of 1498.24, 1510.52, and 1234.15 L·m^−2^·h^−1^ (Figure 5c).

**Figure 6:**
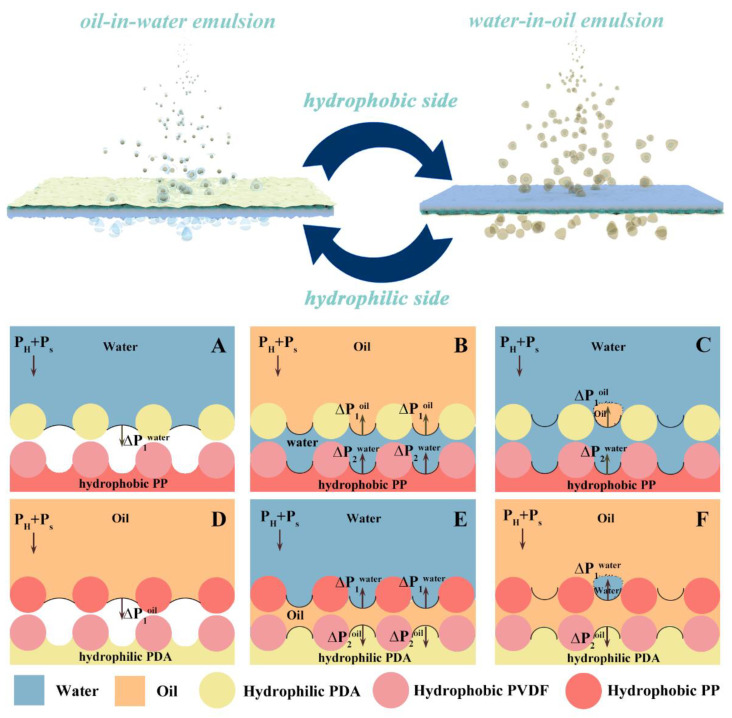
Schematic diagram of mechanism in as-prepared Janus membrane for separating (**A**,**B**) water/light oil mixtures, (**C**) oil-in-water emulsion, (**D**,**E**) heavy oil-water mixtures, and (**F**) water-in-oil emulsion.

The underlying mechanism of separation properties is illuminated in Figure 6. Proverbially, the liquid wetting and osmosis phenomenon occurring during the membrane separation process is correlated to the capillary pressure difference (*∆p*), which can be calculated using the Young-Laplace equation (Equation (3)) [38].

*∆p* = (*2γ_p_ cosθ*)/*R*, (3)

where *γ_p_* is the liquid surface tension, *θ* is the water contact angle or oil contact angle on the surface of the membrane, and R represents the pore radius of the membrane. The positive capillary pressure propels the liquid passing into the materials through inner pores when the membrane is lyophilic (*θ* < 90°). Conversely, the negative capillary pressure will repel the intrusion behavior of a given liquid on the membrane surface if the membrane is lyophobic (*θ* > 90°). In terms of the suction filter separation, the total liquid transmembrane pressure (*P*_total_) is composed of suction force (*P*_s_, provided by vacuum pump), hydrostatic pressure (*P*_H_), and capillary pressure, which can be described by Equations (4) and (5) [39].

*P_total_* = *∆p* + *Ps* + *P_H_*, (4)

*P_H_* = *ρgh*, (5)

where *ρ* refers to the liquid density, g represents the acceleration of gravity, and h is the height of the liquid. Thus, the value of *P*_H_ would gradually decrease due to the separation of the liquid mixture.

During the light oil (hexane)/water mixture or o/w emulsion separation, the hydrophilic side was fixed upward, leading to a positive value of *∆p*^water^ (i.e., *Δp* was downward), and thus facilitated the water permeation process (Figure 6A). When the water accessed the hydrophobic layer, the *∆p*^water^ turned upward accordingly (Figure 6B). Comparatively, the upward capillary force was smaller than the downward suction force; thus, the water could still penetrate the membrane as a function of *P*_s_ and *P*_H_. In terms of the oil component, the PDA/P-PVDA@PP membrane prewetted with water formed elastic hydration layers to resist the oil permeation (Figure 6B,C). Of note, the water viscosity of the hydration layer was greater than that of general water [40]. Its elasticity was similar to a solid, which substantially improved the oil breakthrough pressure of the PDA/P-PVDA@PP membrane. In addition, the underneath PP non-woven fiber played a supporting role in preventing the membrane or pore structure from deforming under a suction force condition, meaning that the hydration layer on the Janus membrane was relatively stable. Therefore, it was hard for the oil components to break through the PDA/P-PVDA@PP membrane with a hydration layer, even under suction conditions.

For the heavy oil-water and o/w emulsion separation, the upward PP side was air-oleophilic so that the oil could easily pass through. As revealed in Figure 6D,E, the capillary force was maintained downward throughout the separation process because the different layers of the PDA/P-PVDA@PP membrane possessed a similar inherent oleophilic nature. On the other hand, the PP side with excellent under-oil hydrophobicity and in-air hydrophobicity provided substantial barriers to withstand the water intrusion (Figure 6F). As a result, high separation ratios (w/o emulsion or mixtures) were achieved in an as-designed PDA/P-PVDA@PP Janus membrane with the PP side facing up.

Additionally, another point should be noted that high-density oil (chloroform, ρ = 1.49 g cm^−3^) possessed a higher flux than the other light oils in the w/o emulsion separation processes while showing the lowest flux as well as a reduced rejection rate for the o/c emulsion. These results suggest that oil density plays a vital role in the oil-water separation process, where the heavier component might easily deposit on the membrane surface during o/w emulsion separation, causing greater transmembrane resistance. Regarding the w/o emulsion separation, to a certain extent, higher density can boost the chloroform to cross the membrane, achieving a much higher flux. In comparison, w/h and w/t emulsions with a low-density oil component may generate buoyancy, leading to a relatively low flux [41].

In addition, the recycling property is also crucial for membranes in practical applications. Therefore, filtration-cleaning cycling experiments were conducted with an as-prepared Janus membrane in the switchable oily emulsion separation process to estimate the recycling performance. As exhibited in Figure 5b,d, the stable fluxes and separation efficiencies were obtained on both sides of the Janus membrane during all six cycles of tests, elucidating its excellent recoverability and reusability. Importantly, compared with previous works [42,43,44,45], the as-designed PDA/P-PVDA@PP Janus membrane exhibited superior performances of emulsions separation, as presented in Table S1, showing great potential in the field of multifunctional emulsion separation (Refs. [21,42,43,44,45] were cited in Supplementary Materials).

## 4. Conclusions

In summary, we developed a novel spacer-assisted VIPS/NIPS method to prepare a hydrophobic P-PVDF@PP substrate membrane, creating a multiscale roughened surface and enhancing underwater oil resistance behavior. Moreover, this surface in the as-designed P-PVDF@PP substrate with affluent active sites effectively facilitated the wet chemical modification process for the obtainment of Janus membranes. Therefore, the as-prepared PDA/P-PVDF@PP Janus membrane with switchable wettability had robust and high-efficiency oil-water separation capacity. In addition, the separation efficiency of the PDA/P-PVDF@PP Janus membrane driven by vacuum pump achieved 99% in most emulsion separation cases, and the reusability of the Janus membrane was desirable. These results demonstrated that the as-designed PDA/P-PVDF@PP Janus membrane could be potentially applied to practical oily wastewater purification. Meanwhile, the as-designed strategy for the fabrication of Janus membranes with asymmetric wettability will inspire the development of Janus oil-water separation membrane systems.

## Data Availability

Data are available on request.

## Supplementary Materials

The following supporting information can be downloaded at: https://www.mdpi.com/article/10.3390/membranes13010014/s1, Figure S1: The illustration of rhombus mesh spacer; Figure S2: The cross-sectional image of the P-PVDF@PP membrane; Figure S3: The water contact angle of (A) the PVDF@PP membrane and (B) the P-PVDF@PP membrane; Figure S4: The water contact angle of (A) PP substrate and (B) PDA/P-PVDF@PP/PDA membrane; Figure S5: Photographs of the surfactant-stabilized (A,B) n-octane-in-water emulsion and the corresponding filtrate, (C,D) water-in-oil emulsion, and the corresponding filtrate; Table S1: Comparison of emulsion separation membrane performance according to different studies.
