# Supplementary material for: Rational Design of PDA/P-PVDF@PP Janus Membrane with Asymmetric Wettability for Switchable Emulsion Separation
Source: Membranes (Basel). 2022 Dec 22;13(1):14. doi: 10.3390/membranes13010014 (PMC9861049; doi:10.3390/membranes13010014)
Supplement: Supplementary file 1 [file membranes-13-00014-s001.zip › membranes-2033832-supplementary.pdf]

membranes

MDPI

Supplementary Material

Rational design of PDA/P−PVDF@PP Janus membrane with asymmetric wettability for switchable emulsion separation

Jingjun Peng [1], Bhaskar Jyoti Deka [2], Shaodi Wu [3], Zhongyuan Luo [1], Jehad A. Kharraz [4,*] and Wei Jia [1,5,*]

[1] National Innovation Center for Advanced Medical Devices, National Institute of Advanced Medical Devices, Shenzhen 518110, China
[2] Department of Hydrology, Indian Institute of Technology Roorkee, Roorkee 247667, India
[3] Shanxi Engineering Research Center of Biorefinery, Institute of Coal Chemistry, Chinese Academy of Sciences, 27 South Taoyuan Road, Taiyuan 030001, China
[4] School of Energy and Environment, City University of Hong Kong, Tat Chee Avenue Kowloon, Hong Kong SAR, China
[5] Institute of Biomedical and Health Engineering, Shenzhen Institute of Advanced Technology, Chinese Academy of Sciences, Shenzhen 518110, China
* Correspondence: wei.jia@nmed.org.cn (W.J.); jehad.kharraz@my.cityu.edu.hk (J.A.K.)

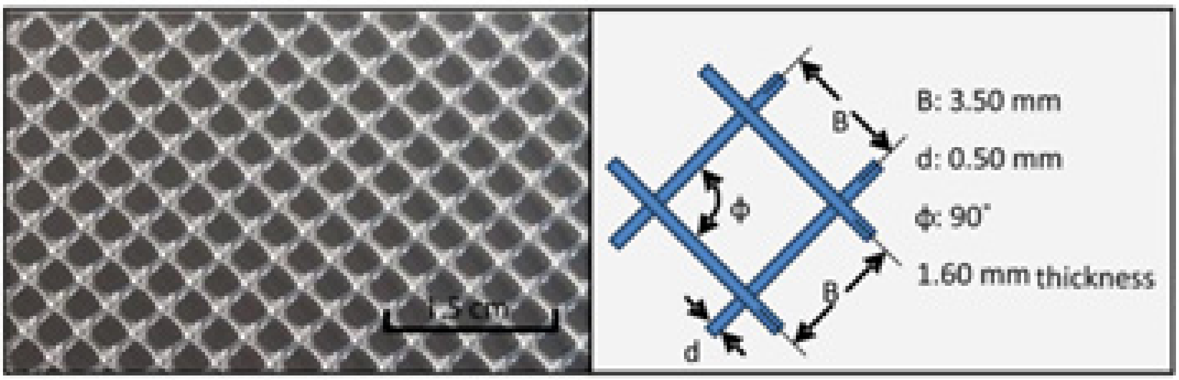

Figure S1. The illustration of rhombus mesh spacer.

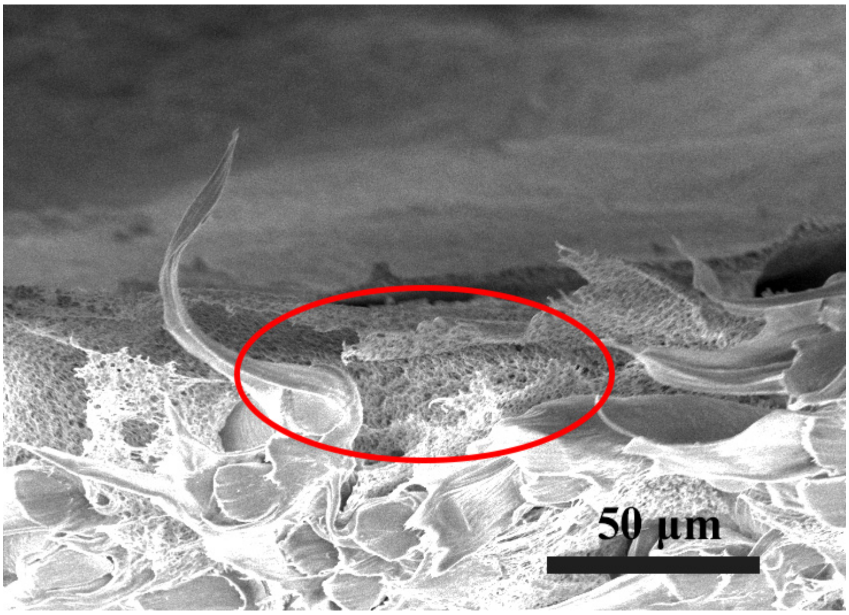

Figure S2. The cross-sectional image of the P−PVDF@PP membrane.

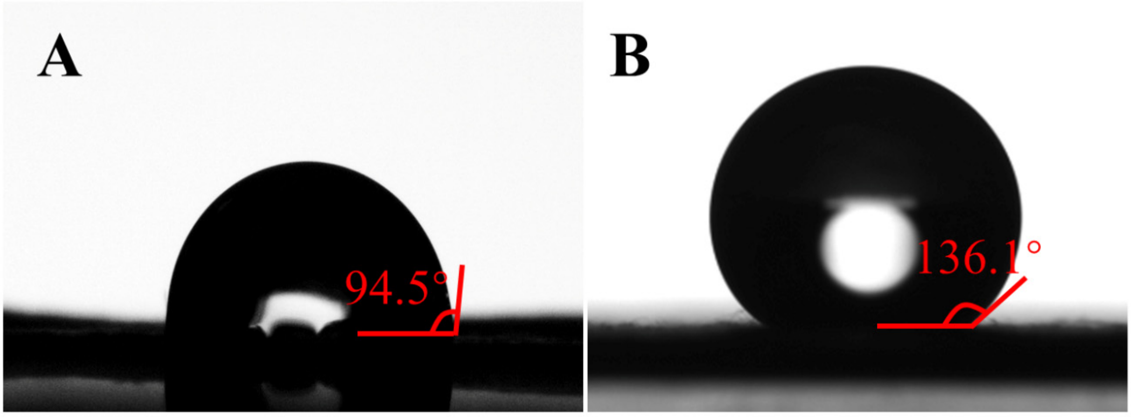

Figure S3. The water contact angle of (A) PVDF@PP membrane and (B) P–PVDF@PP membrane.

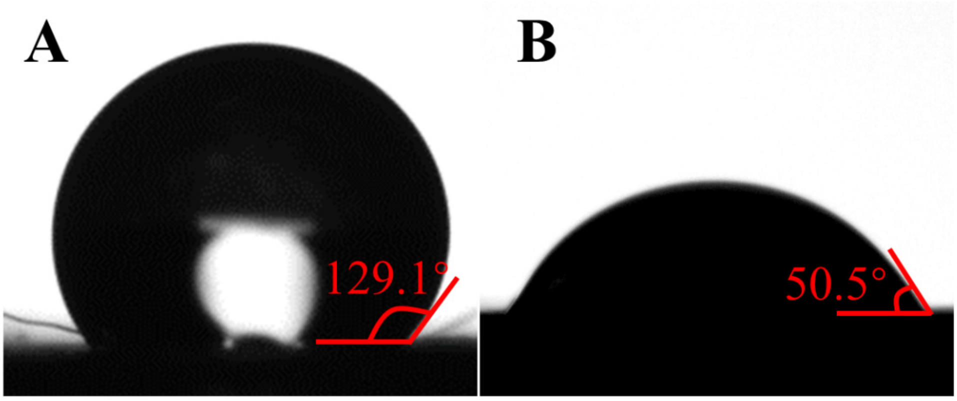

Figure S4. The water contact angle of (A) PP substrate and (B) PDA/P–PVDF@PP/PDA membrane.

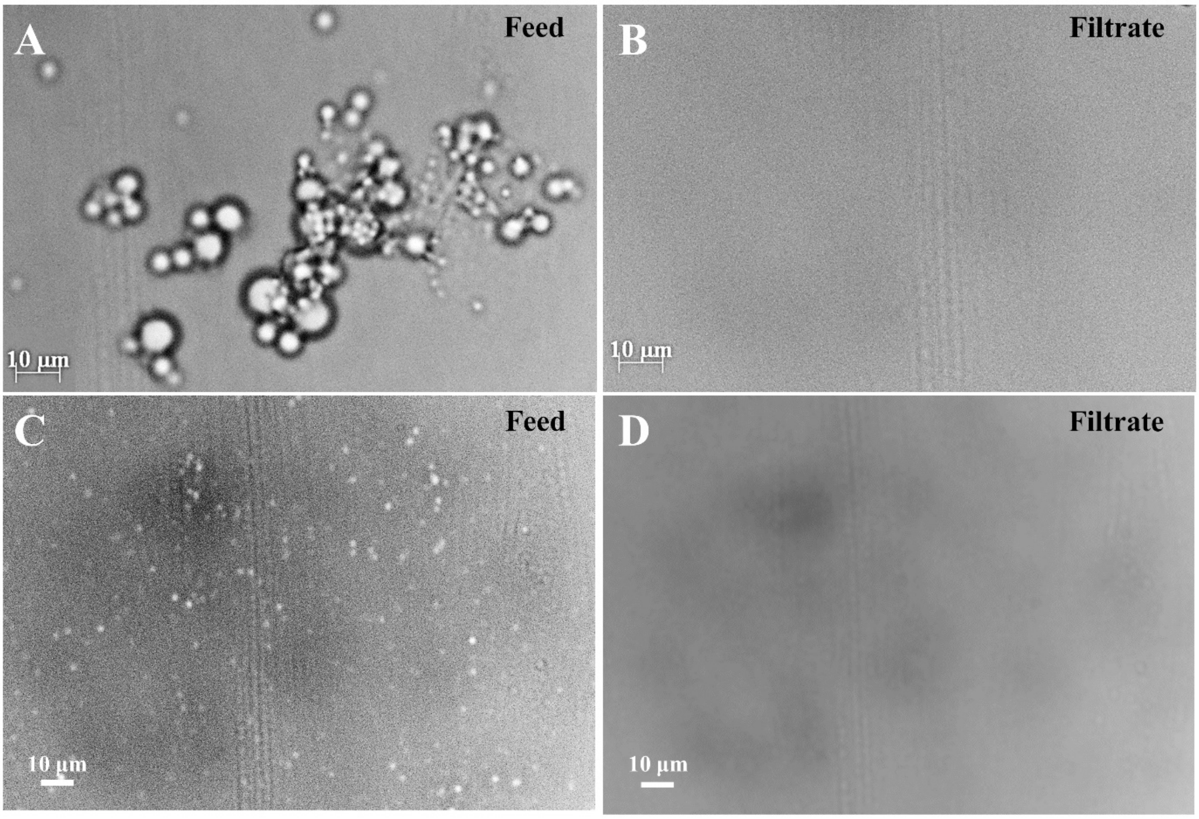

Figure S5. Photographs of the surfactant-stabilized (A-B) n-octane-in-water emulsion and the corresponding filtrate, (C-D) water-in-oil emulsion, and the corresponding filtrate.

Table S1. Comparison of emulsion separation membrane performance with different studies.

Membranes	Method	O/W	W/O	Ref
Janus-–PVDF	Thiol-ene click reaction	$\eta = 97.5\%$	$\eta = 98.5\%$	[1]
PDA/PEI/PVDF/SiO_2	Thermally induced phase separation; spray coating	$\eta \geq 94.0$ %	$\eta \geq 97.0$ %	[2]
Janus F–TiO_2@PPS	Interfacial grafting	$\eta \geq 98.0$ %	$\eta \geq 98.0$ %	[3]
P(GMA-co-mPEGMA)-coated PVDF membrane	Spray-coating	$\eta = 99.0$ %	/	[4]
γ-Fe_2O_3@PVDF/CuO	Chemical oxidation; electrospraying	$\eta \geq 99.0$ %	$\eta \geq 98.5$ %	[5]
PDA/P–PVDF@PP Janus membrane	Spacer-assisted NIPS; in situ deposition; peeling-off	$\eta \geq 95.0$ % (heavy oil); $\eta \geq 99.5$ % (light oil)	$\eta \geq 99.0$ %	This work

References

1. Li, X.; Zhang, W.; Qu, R.; Liu, Y.; Wei, Y.; Feng, L., Asymmetric superwetting configuration of Janus membranes based on thiol–ene clickable silane nanospheres enabling on-demand and energy-efficient oil–water remediation. *J. Mater. Chem. A* **2019,** *7*, 10047-10057.
2. Zuo, J.-H.; Gu, Y.-H.; Wei, C.; Yan, X.; Chen, Y.; Lang, W.-Z., Janus polyvinylidene fluoride membranes fabricated with thermally induced phase separation and spray-coating technique for the separations of both W/O and O/W emulsions. *J. Membrane Sci.* **2020,** *595*, 117475.
3. Yang, C.; Han, N.; Han, C.; Wang, M.; Zhang, W.; Wang, W.; Zhang, Z.; Li, W.; Zhang, X., Design of a Janus F-TiO_2@PPS Porous Membrane with Asymmetric Wettability for Switchable Oil/Water Separation. *ACS Appl. Mater. Inter.* **2019,** *11*, 22408-22418.

4. Xing, J.; Zhang, G.; Jia, X.; Liu, D.; Wyman, I., Preparation of Multipurpose Polyvinylidene Fluoride Membranes via a Spray-Coating Strategy Using Waterborne Polymers. *ACS Appl. Mater. Inter.* **2021,** *13*, 4485-4498.
5. He, H.; Wu, Y.; Wang, Y.; Zhang, T.-J.; Zhang, T. C.; Yuan, S., Constructing A Janus membrane with extremely asymmetric wettability for water unidirectional permeation and switchable emulsion separation. *Sep. Purif. Technol.* **2022,** *303*, 122254.
